# Supplementary figures and images for: Novel decorating behaviour of silk retreats in a challenging habitat
Source: PeerJ. 2022 Mar 22;10:e12839. doi: 10.7717/peerj.12839 (PMC8953501; doi:10.7717/peerj.12839)

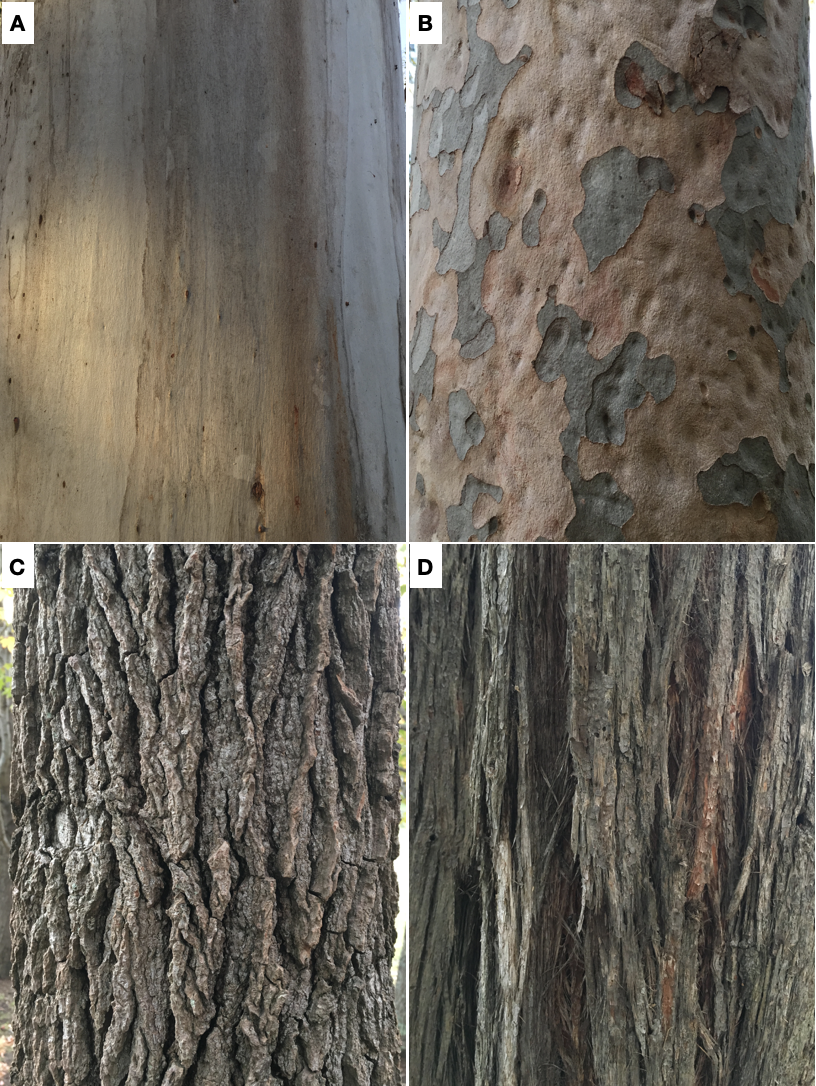

Supplement: Supplemental Information 2 — (A) and (B) show the trees on which Arasia mullion was found, Flooded Gum (Eucalyptus grandis) and Spotted Gum (Corymbia maculata) respectively. (C) and (D) show the surface of trees where no A. mullion spiders were found during the study, Narrow-leaved Iron Bark (Eucalyptus crebra) and Tallowwood (Eucalyptus microcorys) respectively. [file peerj-10-12839-s002.png]
