# Supplementary material for: Novel decorating behaviour of silk retreats in a challenging habitat
Source: PeerJ. 2022 Mar 22;10:e12839. doi: 10.7717/peerj.12839 (PMC8953501; doi:10.7717/peerj.12839)
Supplement: Supplemental Information 3 [file peerj-10-12839-s003.docx]

|  | Name | # Trees | # Retreats | Mean retreats/tree |
| --- | --- | --- | --- | --- |
| 1 | Spotted Gum (*Corymbia maculata*) | 14 | 654 | 46.71 |
| 2 | Smooth-barked Apple Myrtle (*Angophora costata*) | 7 | 148 | 21.14 |
| 3 | Flooded Gum (*Eucalyptus grandis*) | 6 | 142 | 23.67 |
| 4 | Scribbly Gum (*Eucalyptus racemosa*) | 1 | 8 | 8.00 |
| 5 | Argyle Apple (*Eucalyptus cinerea*) | 1 | 0 | 0.00 |
| 6 | Blackbutt (*Eucalyptus pilularis*) | 2 | 0 | 0.00 |
| 7 | Chineses Weeping Elm (*Triadica sebifera*) | 2 | 0 | 0.00 |
| 8 | Forest Red Gum (*Eucalyptus tereticornis*) | 3 | 0 | 0.00 |
| 9 | Hills Weeping Fig (*Ficus microcarpa* var.'Hillii') | 1 | 0 | 0.00 |
| 10 | Illawarra Flame Tree (*Brachychiton acerifolius*) | 2 | 0 | 0.00 |
| 11 | London Plane (*Platanus* *hispanica* ) | 1 | 0 | 0.00 |
| 12 | Moreton Bay Fig (*Ficus macrophylla*) | 1 | 0 | 0.00 |
| 13 | Narrow-leaved Iron Bark (*Eucalyptus crebra*) | 2 | 0 | 0.00 |
| 14 | Queensland Box (*Lophostemon confertus*) | 4 | 0 | 0.00 |
| 15 | River Peppermint (*Eucalyptus elata*) | 1 | 0 | 0.00 |
| 16 | Scotch Elm (*Ulmus glabra*) | 1 | 0 | 0.00 |
| 17 | Southern Blue Gum (*Eucalyptus globulus*) | 3 | 0 | 0.00 |
| 18 | Southern Mahogany (*Eucalyptus botryoides*) | 2 | 0 | 0.00 |
| 19 | Tallowwood (*Eucalyptus microcorys*) | 3 | 0 | 0.00 |
|  |  |  |  |  |
|  | Total tree **species** in the study site | 19 |  |  |
|  | Total trees | 57 |  |  |
|  | Total species **with** *A. mullion*’s retreat | 4 |  |  |
|  | Total species **without** *A. mullion*’s retreat | 15 |  |  |
