# Supplementary material for: Novel decorating behaviour of silk retreats in a challenging habitat
Source: PeerJ. 2022 Mar 22;10:e12839. doi: 10.7717/peerj.12839 (PMC8953501; doi:10.7717/peerj.12839)
Supplement: Supplemental Information 4 [file peerj-10-12839-s004.docx]

| Post Hoc Comparisons - Survey time ✻ Occupancy | | | | | | | | | | | | | | | | | | | | | | | | | | | |  |
| --- | --- | --- | --- | --- | --- | --- | --- | --- | --- | --- | --- | --- | --- | --- | --- | --- | --- | --- | --- | --- | --- | --- | --- | --- | --- | --- | --- | --- |
| **Comparison** | | | | | | | | | | |  | | | | | | | | | | | | | | | | |  |
| **Survey time** | | **Occupancy** | |  | | **Survey time** | | | **Occupancy** | | **Mean Difference** | | | **SE** | | **df** | | | | **t** | | | **p(tukey)** | | | **Cohen's d** | | |
| 2018-07 |  | Vacant |  | - | 2018-07 | |  | Occupied | |  | -0.17498 |  | | 0.031 |  | | | 542 | -5.531 | |  | < .001 | |  | -1.1539 | |  |  |
|  |  |  |  | - | 2018-10 | |  | Vacant | |  | -0.01499 |  | 0.0309 | |  | | 542 | | -0.486 | |  | 1.000 | |  | -0.0989 | |  |  |
|  |  |  |  | - | 2018-10 | |  | Occupied | |  | -0.14099 |  | 0.0439 | |  | | 542 | | -3.212 | |  | 0.062 | |  | -0.9298 | |  |  |
|  |  |  |  | - | 2019-01 | |  | Vacant | |  | 0.11952 |  | 0.0397 | |  | | 542 | | 3.012 | |  | 0.108 | |  | 0.7882 | |  |  |
|  |  |  |  | - | 2019-01 | |  | Occupied | |  | 0.14244 |  | 0.0301 | |  | | 542 | | 4.727 | |  | < .001 | |  | 0.9393 | |  |  |
|  |  |  |  | - | 2019-03 | |  | Vacant | |  | 0.10441 |  | 0.0339 | |  | | 542 | | 3.079 | |  | 0.090 | |  | 0.6885 | |  |  |
|  |  |  |  | - | 2019-03 | |  | Occupied | |  | -0.01935 |  | 0.0333 | |  | | 542 | | -0.581 | |  | 1.000 | |  | -0.1276 | |  |  |
|  |  |  |  | - | 2019-06 | |  | Vacant | |  | 0.04196 |  | 0.0315 | |  | | 542 | | 1.332 | |  | 0.975 | |  | 0.2767 | |  |  |
|  |  |  |  | - | 2019-06 | |  | Occupied | |  | -0.22335 |  | 0.0301 | |  | | 542 | | -7.431 | |  | < .001 | |  | -1.4729 | |  |  |
|  |  |  |  | - | 2019-10 | |  | Vacant | |  | -0.08999 |  | 0.0308 | |  | | 542 | | -2.926 | |  | 0.135 | |  | -0.5934 | |  |  |
|  |  |  |  | - | 2019-10 | |  | Occupied | |  | -0.26459 |  | 0.0415 | |  | | 542 | | -6.371 | |  | < .001 | |  | -1.7449 | |  |  |
|  |  | Occupied |  | - | 2018-10 | |  | Vacant | |  | 0.15998 |  | 0.0283 | |  | | 542 | | 5.647 | |  | < .001 | |  | -1.0550 | |  |  |
|  |  |  |  | - | 2018-10 | |  | Occupied | |  | 0.03398 |  | 0.0422 | |  | | 542 | | 0.806 | |  | 1.000 | |  | 0.2241 | |  |  |
|  |  |  |  | - | 2019-01 | |  | Vacant | |  | 0.29450 |  | 0.0378 | |  | | 542 | | 7.800 | |  | < .001 | |  | -1.9421 | |  |  |
|  |  |  |  | - | 2019-01 | |  | Occupied | |  | 0.31741 |  | 0.0276 | |  | | 542 | | 11.521 | |  | < .001 | |  | 2.0933 | |  |  |
|  |  |  |  | - | 2019-03 | |  | Vacant | |  | 0.27938 |  | 0.0316 | |  | | 542 | | 8.832 | |  | < .001 | |  | -1.8425 | |  |  |
|  |  |  |  | - | 2019-03 | |  | Occupied | |  | 0.15562 |  | 0.0310 | |  | | 542 | | 5.021 | |  | < .001 | |  | 1.0263 | |  |  |
|  |  |  |  | - | 2019-06 | |  | Vacant | |  | 0.21694 |  | 0.0290 | |  | | 542 | | 7.468 | |  | < .001 | |  | -1.4307 | |  |  |
|  |  |  |  | - | 2019-06 | |  | Occupied | |  | -0.04837 |  | 0.0275 | |  | | 542 | | -1.761 | |  | 0.838 | |  | -0.3190 | |  |  |
|  |  |  |  | - | 2019-10 | |  | Vacant | |  | 0.08499 |  | 0.0282 | |  | | 542 | | 3.011 | |  | 0.108 | |  | -0.5605 | |  |  |
|  |  |  |  | - | 2019-10 | |  | Occupied | |  | -0.08961 |  | 0.0397 | |  | | 542 | | -2.258 | |  | 0.508 | |  | -0.5910 | |  |  |
| 2018-10 |  | Vacant |  | - | 2018-10 | |  | Occupied | |  | -0.12600 |  | 0.0416 | |  | | 542 | | -3.030 | |  | 0.103 | |  | -0.8309 | |  |  |
|  |  |  |  | - | 2019-01 | |  | Vacant | |  | 0.13452 |  | 0.0371 | |  | | 542 | | 3.625 | |  | 0.016 | |  | 0.8871 | |  |  |
|  |  |  |  | - | 2019-01 | |  | Occupied | |  | 0.15743 |  | 0.0266 | |  | | 542 | | 5.908 | |  | < .001 | |  | 1.0382 | |  |  |
|  |  |  |  | - | 2019-03 | |  | Vacant | |  | 0.11940 |  | 0.0309 | |  | | 542 | | 3.870 | |  | 0.007 | |  | 0.7874 | |  |  |
|  |  |  |  | - | 2019-03 | |  | Occupied | |  | -0.00436 |  | 0.0302 | |  | | 542 | | -0.144 | |  | 1.000 | |  | -0.0287 | |  |  |
|  |  |  |  | - | 2019-06 | |  | Vacant | |  | 0.05696 |  | 0.0282 | |  | | 542 | | 2.020 | |  | 0.680 | |  | 0.3756 | |  |  |
|  |  |  |  | - | 2019-06 | |  | Occupied | |  | -0.20835 |  | 0.0266 | |  | | 542 | | -7.845 | |  | < .001 | |  | -1.3741 | |  |  |
|  |  |  |  | - | 2019-10 | |  | Vacant | |  | -0.07499 |  | 0.0273 | |  | | 542 | | -2.742 | |  | 0.209 | |  | -0.4946 | |  |  |
|  |  |  |  | - | 2019-10 | |  | Occupied | |  | -0.24959 |  | 0.0391 | |  | | 542 | | -6.388 | |  | < .001 | |  | -1.6460 | |  |  |
|  |  | Occupied |  | - | 2019-01 | |  | Vacant | |  | 0.26052 |  | 0.0485 | |  | | 542 | | 5.371 | |  | < .001 | |  | -1.7180 | |  |  |
|  |  |  |  | - | 2019-01 | |  | Occupied | |  | 0.28343 |  | 0.0411 | |  | | 542 | | 6.903 | |  | < .001 | |  | 1.8692 | |  |  |
|  |  |  |  | - | 2019-03 | |  | Vacant | |  | 0.24540 |  | 0.0439 | |  | | 542 | | 5.590 | |  | < .001 | |  | -1.6184 | |  |  |
|  |  |  |  | - | 2019-03 | |  | Occupied | |  | 0.12164 |  | 0.0434 | |  | | 542 | | 2.800 | |  | 0.183 | |  | 0.8022 | |  |  |
|  |  |  |  | - | 2019-06 | |  | Vacant | |  | 0.18296 |  | 0.0421 | |  | | 542 | | 4.348 | |  | < .001 | |  | -1.2066 | |  |  |
|  |  |  |  | - | 2019-06 | |  | Occupied | |  | -0.08236 |  | 0.0410 | |  | | 542 | | -2.009 | |  | 0.687 | |  | -0.5431 | |  |  |
|  |  |  |  | - | 2019-10 | |  | Vacant | |  | 0.05101 |  | 0.0415 | |  | | 542 | | 1.229 | |  | 0.987 | |  | -0.3364 | |  |  |
|  |  |  |  | - | 2019-10 | |  | Occupied | |  | -0.12360 |  | 0.0500 | |  | | 542 | | -2.471 | |  | 0.361 | |  | -0.8151 | |  |  |
| 2019-01 |  | Vacant |  | - | 2019-01 | |  | Occupied | |  | 0.02291 |  | 0.0365 | |  | | 542 | | 0.628 | |  | 1.000 | |  | 0.1511 | |  |  |
|  |  |  |  | - | 2019-03 | |  | Vacant | |  | -0.01512 |  | 0.0397 | |  | | 542 | | -0.381 | |  | 1.000 | |  | -0.0997 | |  |  |
|  |  |  |  | - | 2019-03 | |  | Occupied | |  | -0.13887 |  | 0.0392 | |  | | 542 | | -3.545 | |  | 0.022 | |  | -0.9158 | |  |  |
|  |  |  |  | - | 2019-06 | |  | Vacant | |  | -0.07756 |  | 0.0377 | |  | | 542 | | -2.060 | |  | 0.652 | |  | -0.5115 | |  |  |
|  |  |  |  | - | 2019-06 | |  | Occupied | |  | -0.34287 |  | 0.0364 | |  | | 542 | | -9.408 | |  | < .001 | |  | -2.2612 | |  |  |
|  |  |  |  | - | 2019-10 | |  | Vacant | |  | -0.20951 |  | 0.0370 | |  | | 542 | | -5.659 | |  | < .001 | |  | -1.3817 | |  |  |
|  |  |  |  | - | 2019-10 | |  | Occupied | |  | -0.38411 |  | 0.0464 | |  | | 542 | | -8.285 | |  | < .001 | |  | -2.5331 | |  |  |
|  |  | Occupied |  | - | 2019-03 | |  | Vacant | |  | -0.03803 |  | 0.0301 | |  | | 542 | | -1.262 | |  | 0.983 | |  | 0.2508 | |  |  |
|  |  |  |  | - | 2019-03 | |  | Occupied | |  | -0.16179 |  | 0.0295 | |  | | 542 | | -5.492 | |  | < .001 | |  | -1.0670 | |  |  |
|  |  |  |  | - | 2019-06 | |  | Vacant | |  | -0.10047 |  | 0.0274 | |  | | 542 | | -3.666 | |  | 0.014 | |  | 0.6626 | |  |  |
|  |  |  |  | - | 2019-06 | |  | Occupied | |  | -0.36579 |  | 0.0257 | |  | | 542 | | -14.220 | |  | < .001 | |  | -2.4123 | |  |  |
|  |  |  |  | - | 2019-10 | |  | Vacant | |  | -0.23242 |  | 0.0265 | |  | | 542 | | -8.759 | |  | < .001 | |  | 1.5328 | |  |  |
|  |  |  |  | - | 2019-10 | |  | Occupied | |  | -0.40702 |  | 0.0385 | |  | | 542 | | -10.570 | |  | < .001 | |  | -2.6842 | |  |  |
| 2019-03 |  | Vacant |  | - | 2019-03 | |  | Occupied | |  | -0.12376 |  | 0.0333 | |  | | 542 | | -3.715 | |  | 0.012 | |  | -0.8162 | |  |  |
|  |  |  |  | - | 2019-06 | |  | Vacant | |  | -0.06244 |  | 0.0315 | |  | | 542 | | -1.982 | |  | 0.706 | |  | -0.4118 | |  |  |
|  |  |  |  | - | 2019-06 | |  | Occupied | |  | -0.32776 |  | 0.0301 | |  | | 542 | | -10.905 | |  | < .001 | |  | -2.1615 | |  |  |
|  |  |  |  | - | 2019-10 | |  | Vacant | |  | -0.19439 |  | 0.0308 | |  | | 542 | | -6.321 | |  | < .001 | |  | -1.2820 | |  |  |
|  |  |  |  | - | 2019-10 | |  | Occupied | |  | -0.36900 |  | 0.0415 | |  | | 542 | | -8.886 | |  | < .001 | |  | -2.4334 | |  |  |
|  |  | Occupied |  | - | 2019-06 | |  | Vacant | |  | 0.06132 |  | 0.0309 | |  | | 542 | | 1.986 | |  | 0.703 | |  | -0.4044 | |  |  |
|  |  |  |  | - | 2019-06 | |  | Occupied | |  | -0.20400 |  | 0.0294 | |  | | 542 | | -6.943 | |  | < .001 | |  | -1.3453 | |  |  |
|  |  |  |  | - | 2019-10 | |  | Vacant | |  | -0.07063 |  | 0.0301 | |  | | 542 | | -2.347 | |  | 0.444 | |  | 0.4658 | |  |  |
|  |  |  |  | - | 2019-10 | |  | Occupied | |  | -0.24524 |  | 0.0410 | |  | | 542 | | -5.975 | |  | < .001 | |  | -1.6173 | |  |  |
| 2019-06 |  | Vacant |  | - | 2019-06 | |  | Occupied | |  | -0.26531 |  | 0.0273 | |  | | 542 | | -9.710 | |  | < .001 | |  | -1.7497 | |  |  |
|  |  |  |  | - | 2019-10 | |  | Vacant | |  | -0.13195 |  | 0.0281 | |  | | 542 | | -4.698 | |  | < .001 | |  | -0.8702 | |  |  |
|  |  |  |  | - | 2019-10 | |  | Occupied | |  | -0.30655 |  | 0.0396 | |  | | 542 | | -7.742 | |  | < .001 | |  | -2.0216 | |  |  |
|  |  | Occupied |  | - | 2019-10 | |  | Vacant | |  | 0.13336 |  | 0.0264 | |  | | 542 | | 5.043 | |  | < .001 | |  | -0.8795 | |  |  |
|  |  |  |  | - | 2019-10 | |  | Occupied | |  | -0.04124 |  | 0.0384 | |  | | 542 | | -1.073 | |  | 0.996 | |  | -0.2720 | |  |  |
| 2019-10 |  | Vacant |  | - | 2019-10 | |  | Occupied | |  | -0.17460 |  | 0.0390 | |  | | 542 | | -4.478 | |  | < .001 | |  | -1.1515 | |  |  |
| *Note.* Comparisons are based on estimated marginal means | | | | | | | | | | | | | | | | | | | | | | | | | | | |  |
|  | | | | | | | | | | | | | | | | | | | | | | | | | | | |  |
