# Supplementary material for: Novel decorating behaviour of silk retreats in a challenging habitat
Source: PeerJ. 2022 Mar 22;10:e12839. doi: 10.7717/peerj.12839 (PMC8953501; doi:10.7717/peerj.12839)
Supplement: Supplemental Information 5 [file peerj-10-12839-s005.docx]

| Tukey Post-Hoc Test – log retreat length | | | | | | | | | | | |
| --- | --- | --- | --- | --- | --- | --- | --- | --- | --- | --- | --- |
|  | |  | | **2019-01** | | **2019-03** | | **2019-06** | | **2019-10** | |
| 2019-01 | | Mean difference |  | — |  | -0.109 | * | -0.327 | *** | -0.3440 | *** |
|  |  | p-value |  | — |  | 0.027 |  | < .001 |  | < .001 |  |
| 2019-03 | | Mean difference |  |  |  | — |  | -0.218 | *** | -0.2349 | *** |
|  |  | p-value |  |  |  | — |  | < .001 |  | < .001 |  |
| 2019-06 | | Mean difference |  |  |  |  |  | — |  | -0.0169 |  |
|  |  | p-value |  |  |  |  |  | — |  | 0.969 |  |
| 2019-10 | | Mean difference |  |  |  |  |  |  |  | — |  |
|  |  | p-value |  |  |  |  |  |  |  | — |  |
| Note. * p < .05, ** p < .01, *** p < .001 | | | | | | | | | | | |
