# Supplementary material for: Novel decorating behaviour of silk retreats in a challenging habitat
Source: PeerJ. 2022 Mar 22;10:e12839. doi: 10.7717/peerj.12839 (PMC8953501; doi:10.7717/peerj.12839)
Supplement: Supplemental Information 6 [file peerj-10-12839-s006.docx]

| Change in retreat size (length) - Pearson's Chi-squared test | | | | | |
| --- | --- | --- | --- | --- | --- |
|  |  |  |  |  |  |
| From survey 1 (Jul 2018) to 2 (Oct 2018) | | | | | |
| X-squared = 0.41004, df = 3, p-value = 0.9382 | | | | | |
|  | | | | | |
|  |  |  |  | Size change |  |
| Occupancy change | | |  | Decrease | Increase |
| Occupied | → | Occupied |  | not significantly different | not significantly different |
| Occupied | → | Vacant |  | not significantly different | not significantly different |
| Vacant | → | Occupied |  | not significantly different | not significantly different |
| Vacant | → | Vacant |  | not significantly different | not significantly different |
|  |  |  |  |  |  |
| From survey 2 (Oct 2018) to 3 (Jan 2019) | | | | | |
| X-squared = 5, df = 3, p-value = 0.1718 | | | | | |
|  | | | | | |
|  |  |  |  | Size change |  |
| Occupancy change | | |  | Decrease | Increase |
| Occupied | → | Occupied |  | not significantly different | not significantly different |
| Occupied | → | Vacant |  | not significantly different | not significantly different |
| Vacant | → | Occupied |  | not significantly different | not significantly different |
| Vacant | → | Vacant |  | not significantly different | not significantly different |
|  |  |  |  |  |  |
| From survey 3 (Jan 2019) to 4 (Mar 2019) | | | | | |
| X-squared = 5.0524, df = 3, p-value = 0.168 | | | | | |
|  | | | | | |
|  |  |  |  | Size change |  |
| Occupancy change | | |  | Decrease | Increase |
| Occupied | → | Occupied |  | not significantly different | not significantly different |
| Occupied | → | Vacant |  | not significantly different | not significantly different |
| Vacant | → | Occupied |  | not significantly different | not significantly different |
| Vacant | → | Vacant |  | not significantly different | not significantly different |
|  |  |  |  |  |  |
| From survey 4 (Mar 2019) to 5 (Jun 2019) | | | | | |
| X-squared = 1.8882, df = 3, p-value = 0.5959 | | | | | |
|  | | | | | |
|  |  |  |  | Size change |  |
| Occupancy change | | |  | Decrease | Increase |
| Occupied | → | Occupied |  | not significantly different | not significantly different |
| Occupied | → | Vacant |  | not significantly different | not significantly different |
| Vacant | → | Occupied |  | not significantly different | not significantly different |
| Vacant | → | Vacant |  | not significantly different | not significantly different |
|  |  |  |  |  |  |
| From survey 5 (Jun 2019) to 6 (Oct 2019) | | | | | |
| X-squared = 1.472, df = 3, p-value = 0.6888 | | | | | |
|  | | | | | |
|  |  |  |  | Size change |  |
| Occupancy change | | |  | Decrease | Increase |
| Occupied | → | Occupied |  | not significantly different | not significantly different |
| Occupied | → | Vacant |  | not significantly different | not significantly different |
| Vacant | → | Occupied |  | not significantly different | not significantly different |
| Vacant | → | Vacant |  | not significantly different | not significantly different |
|  |  |  |  |  |  |
